# 

**Published:** 1891-01

**Authors:** 


### LIST OF CONTRIBUTORS TO VOLUME XII.

Allan, Geo. S., D.D.S.
Andrews, R. R., D.D.S.
Atkinson, Charles B., D.D.S.
Barker, William, D.D.S.
Bartholomew, Dr. S. B.
Beale, Alonzo P., D.D.S.
Boardman, Waldo E., D.M.D.
Brackett, Dr. C. A.
Briggs, Edward C., M.D., D.M.D.
Chupein, Dr. Theodore F.
Clapp, Dwight M., D.M.D.
Curtis, G. Lenox M.D., D.D.S.
Delavan, Dr. D. Bryson.
Dixon, Lewis S., M.D.
Dwinelle, W. H., M.D., D.D.S.
Eames, George F., M.D., D.D.S.
Eisenbrey, J. Lehman, D.D.S.
Ernst, Harold C., M.D.
Farrar, J. N., M.D., D.D.S.
Faught, L. Ashley, D.D.S.
Fillebrown, Thomas, M.D., D.M.D.
Fletcher, T. A., D.D.S.
Friedenwald, B. D., D.D.S.
Giblin, Thomas J., D.M.D.
Grady, Richard, M.D., D.D.S.
Harrison, Walter, D.D.S., D.M.D.
Heitzmann, Carl.
Ingersoll, Dr. L. C.
Junkerman, G. S., M.D., D.D.S.
Knight, Jos. King, D.D.S.
Leffmann, Henry, M.D., D.D.S.
Libbey, J. A., D.D.S.
Marshall, John S., M.D.
Meriam, Horatio C., D.M.D.
Miller, W. D., M.D., D.D.S.
Newton, Richard Cole, M.D.
Niles, Edward S., D.M.D.
Palmer, Dr. S. B.
Perrin, Frank, D.M.D.
Potter, Dr. William H.
Rollins, Dr. Wm. H.
Ross, Dr. A. M.
Schumann, H. H., D.D.S.
Stanton, J. E., M.D., D.M.D.
Stebbins, Dr. E. A.
Stevens, Geo. T., M.D.
Stone, Arthur K., A.M., M.D.
Talbot, Eugene S., M.D., D.D.S.
Terry, Dr. C. T.
Thompson, Alton H., D.D.S.
Townsend, Dr. A. F.
Trueman, William H., D.D.S.
Tucker, E. G., M.D.
Weld, George W., M.D., D.D.S.
Werner, J. G. W., D.M.D.
Wetzel, Dr. Eugene J.
Whitefield, George W., M.D., D.D.S.
Wight, J. S., M.D.
Williams, Jacob L., M.D.
Woodburn, J. Cowan, M.D.

